# Identification of co-regulated transcripts affecting male body size in *Drosophila*

**DOI:** 10.1186/gb-2005-6-6-r53

**Published:** 2005-06-01

**Authors:** Cynthia J Coffman, Marta L Wayne, Sergey V Nuzhdin, Laura A Higgins, Lauren M McIntyre

**Affiliations:** 1 Health Services Research and Development Biostatistics Unit, Durham VA Medical Center (152), Durham, NC 27705, USA; 2 Duke University Medical Center, Department of Biostatistics and Bioinformatics, Durham, NC 27710, USA; 3 Department of Zoology, University of Florida, Gainesville, FL 32611, USA; 4 Department Ecology and Evolution, University of California at Davis, Davis, CA 95616, USA; 5 Department of Agronomy, Purdue University, West Lafayette, IN 47907, USA

## Abstract

Factor analysis is an analytic approach that describes the covariation among a set of genes through the estimation of 'factors', which may be, for example, transcription factors, microRNAs (miRNAs), and so on, by which the genes are co-regulated. Factor analysis gives a direct mechanism by which to relate gene networks to complex traits. Using simulated data, we found that factor analysis clearly identifies the number and structure of factors and outperforms hierarchical cluster analysis. Noise genes, genes that are not correlated with any factor, can be distinguished even when factor structure is complex. Applied to body size in *Drosophila simulans*, an evolutionarily important complex trait, a factor was directly associated with body size.

## Abstract 2

Factor analysis is applied to microarray data in order to relate gene networks to complex traits and identifies a factor associated with body size in * Drosophila simulans*.

## Background

Unraveling complex traits requires an understanding of how genetic variation results in variation among transcript levels, proteins, and metabolites, and how this variation generates phenotypic variation. These distinct levels in the biological system are interdependent. The ability to model interactions among loci at each of these levels, and relationships between levels, is key to providing insight into complex traits. The promise of genomic and proteomic technology is in capturing variation for thousands of loci simultaneously. This affords an unprecedented opportunity to understand the consequences of genetic variation. Many studies have exploited this ability through the use of mutant analysis applied to whole-genome transcript arrays. Mutant analysis provides insight into the impact of a mutation on a gene network and whole-genome studies of transcription have revealed misexpression due to gene knockouts and have established redundancy and specificity of transcriptional regulation [1]. Cluster analysis has been successfully combined with tests of differential expression to study whole-genome response to mutation in order to develop hypotheses about co-regulation and coordinated expression [2,3].

However, the consequences of such strong perturbations are difficult to apply to pathways in non-mutant individuals. In addition, the mutations chosen usually cause a severe alteration in a single gene, such as a knockout. Natural variants introduce smaller changes in pathways [4] and natural variants may exhibit allelic differences at several loci. Natural variation in the transcriptome as a consequence of genetic variation has been demonstrated [5,6]. Natural genotypes can also be mated in a deliberate manner and the progeny of such matings can be used to estimate the genetic architecture of individual traits [7,8], and to link traits across different levels of the biological system [9,10]. We focus here on providing insight into how coordinated gene expression affects phenotype. Links between transcript abundance and phenotypic variation have been established [11-15]. What is needed now is an analytic approach that allows interpretation of the relationships among transcript levels and modeling of the link between transcript level and complex trait.

Factor analysis is an analytic approach that describes the covariation among a set of genes through the estimation of factors. One may interpret the factor as the mechanism, for example transcription factors, microRNAs (miRNAs), and so on, by which genes are co-regulated. The resulting factor model represents sets of coordinately expressed genes. Genes may participate in multiple factors. Principal components analysis, spectral map analysis and correspondence analysis are alternative multivariate techniques for microarray analysis [16,17] that can all be used in this capacity. Factor analysis, however, provides a convenient representation of the gene network by describing each gene's association with the factor as a load (between -1 and 1), where the strength of the load indicates how much influence the transcript level of that gene has upon the factor. The factor can then be examined for associations with complex traits [18]. Factor analysis is the extension of Sewell Wright's work on the correspondence among traits [19], and as such is perfectly suited for modeling the relationships among transcript levels for a set of crosses. The high dimensionality of genome-wide expression data presents special challenges. This challenge, primarily the ill-conditioned matrices resulting from such studies, has been well described and explicitly acknowledged in much of the literature on the analysis of gene-expression data [20-22]. If thousands of genes belonging to dozens of networks are simultaneously considered as current theory indicates, spurious associations may emerge and/or true associations may be obscured [23,24]. Previous applications of factor analyses to array data [25,26] dealt with this issue by an initial reduction of dimensionality through the use of cluster analysis.

Using simulation studies, we evaluate the utility of factor analysis for identifying covariation in gene-expression data and identifying underlying factors. We compare the performance of factor analysis to hierarchical clustering and tight clustering [27]. We then test the estimation of factors on a set of *Drosophila *lines for genes involved in the immune pathway. The immune system provides a relatively well understood set of interactions and as such allows a real data check on the applicability of factor analysis to microarray data.

A logical next step is to use factor analysis to relate variation among transcript levels to phenotypic variation, a step not possible in a cluster analysis. For *Drosophila*, body size is a complex trait where latitudinal clines in body size have been repeatedly demonstrated across ectotherms [28]. In *D. subobscura*, a body-size cline evolved in 12 decades, thus ranking body size in flies among the fastest-evolving morphological traits ever observed in natural populations [29]. The proximate reasons for these clines are complex, especially given that body size in flies is positively correlated with mating success in males [30-32]. Of further interest are data suggesting that the same genomic regions are involved in adaptation in two of these clines, South America and Australia [33]. In contrast to the immune system, there is little *a priori *information on how the candidates genes are related to one another. In addition, identification of factors associated with variation in body size in natural populations of *Drosophila *is a question of great evolutionary interest.

## Results

### Simulations

In the initial scenario, a sample size of 100 individuals was examined. This sample size is large for a microarray experiment, but is in the low range of the minimum sample size suggested in factor analysis methodology [34]. We simulated a high degree of correlation among genes within a factor (ρ = 0.80), three factors with a manageable number of genes associated within each factor (correlated genes: 30), and some genes not associated with any factor (noise genes: 100). We assume genetically variable lines for which differences in transcript abundance among lines was moderate within each of the three factors [35]. Factor analysis on these data was performed. Factors were identified by examining the eigenvalues of the correlation matrix [23]. The first five eigenvalues were 25.3, 21.3, 18.3, 4.3, and 4.1. The substantial drop between the third and fourth eigenvalues (from 18.3 to 4.3) indicates that three factors (the number simulated) are clearly identified, explaining 34% of the variation. We then set the number of factors in the analysis to three, and estimated factor loadings in order to examine the structure of the factor. All (100%, *n *= 90) of the correlated genes loaded [36] on the correct factor, with none of the noise genes loading on any factor (see Table 1). Reducing the correlation among factors, and reducing the effect size do not affect the ability of factor analysis to identify the correct underlying structure (Table 1).

Results of a hierarchical cluster analysis found that the three groups of genes clustered together with the noise genes which formed two distinct clusters. However, discriminating the true clusters from the noise clusters was not obvious using standard approaches. Tight clustering [27], where a resampling strategy is used to separate noise genes from signal, on these data was interesting. If the number of clusters is set to the true value of three, all 190 genes are identified as noise. If the number of clusters is set to five, 45 of the 100 noise genes are correctly identified as noise. All of the correlated genes are placed into the correct clusters. The remaining 55 noise genes are placed into clusters.

**Figure 1:**
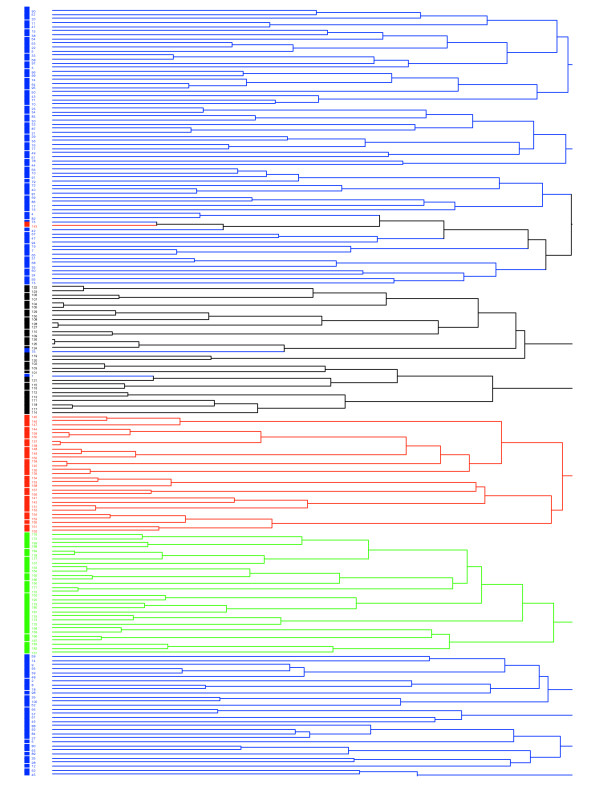
Hierarchical cluster plot of simulation with two genotypes, 100 noise genes, and three factors. ρ = 0.40, effect size = 0.2, 0.4, and 0.6. Blue, noise genes; green, genes from underlying factor 1; red, genes from underlying factor 2; black, genes from underlying factor 3.

For the case with lower effect size and lower correlation, the dendrogram resulting from hierarchical cluster analysis is given in Figure 1. As in the factor analysis, the three groups of genes clustered together well, although not perfectly. Once again, however, statistics for determining the appropriate number of clusters did not clearly identify the correct number of clusters. The noise genes also seem to follow discernible clustering patterns. In tight clustering, when the correct number of clusters are specified and the number of extra clusters (*k*0) is set to 6-7, 23 of the 90 correlated genes are identified as noise and all of the noise genes are correctly identified. Setting the number of clusters higher results in clusters of noise genes. In this simple case, factor analysis clearly outperforms both traditional hierarchical cluster analysis and tight clustering, as it is easily able to discern the correct number of underlying clusters.

We then increased the number of genes from a total of 190 (90 in the three networks and 100 noise genes) to 1,900 (900 in the three factors and 1,000 noise genes). Using factor analysis, we easily identified the correct number of factors and 100% of the genes in each factor loaded on the correct factor (see Table 1). Lowering the correlation among genes in a factor to ρ = 0.4 resulted in the reduction of the explanatory power of the factor analysis. The number of underlying factors was correctly identified although, as expected, the total variation explained by the factors was reduced. Of the correlated genes, 66% loaded on the correct factors and only one noise gene (out of 1,000) was mistakenly placed into a factor. Given the reasonable fractions identified when the number of genes in factors differs by an order of 10 (190 versus 1,900), and the fact that our recovery of the structure was virtually unchanged, it is apparent that the number of genes in a factor does not impact on the ability of factor analysis to recover the factor structure. In contrast, hierarchical cluster analysis performs less well as the number of noise genes increases, with the noise genes increasing in their dispersion among clusters.

In a set of simulations to match our *Drosophila *experimental design, 10 genotypes with three replicates per genotype for a total of 30 samples (chips) were simulated. Averaging transcript abundance within each genotype removed uninteresting variation and increased resolution (data not shown). We began with three factors of 30 genes each, and 100 noise genes. The maximum difference in transcript abundance among genotypes was large (1, 2 and 3). In this case, the three factors were not clearly identified. To determine whether genes would be correctly placed inside factors, the number of factors was set to three. When difference in transcript abundance among genotypes was lowest, fewer genes were placed in the correct factor (13/30). On the other hand, where difference in transcript abundance among genotypes was highest and 100% of the correlated genes loaded on the correct factor, 29 of the genes from the other two factors and 29 noise genes were erroneously identified as a part of this factor. When correlation among genes within a factor was reduced to ρ = 0.40, the difficulty in identifying genes on the correct factor increased.

We hypothesized that the presence of many noise genes, coincident with a sample size of ten, was responsible for the difficulty in identifying the correct number of gene factors. Without the noise genes, when correlation was ρ = 0.8, each of the underlying factors was clearly identified. All (100%) of the genes were correctly placed in the corresponding factors, although several genes were placed in multiple factors. When correlation among genes in a factor was reduced to ρ = 0.40, the number of factors was underestimated and many of the genes were identified in multiple factors. This indicates a complex interplay between the difference in transcript abundance among genotypes, the sample size, the number of noise genes and the correlation structure in the identification of factors.

We also examined the impact of increasing the number of factors beyond the number of genotypes. We simulated a set of 600 genes belonging to 20 factors, with 30 genes in each factor and 100 noise genes. In an analysis with nine factors (the maximum estimable with ten genotypes), factor 1 seemed to capture the majority of all the genes in the 20 factors. When effect size was large, the majority of the noise genes (58%) were identified by their failure to load highly (greater than 0.7) on any factor, and the majority of genes that were correlated did load highly on at least one factor (96%). Lowering the correlation lowers the ability to identify correlated genes as loading highly. In this case, 30% are identified and approximately the same fraction of noise genes (60%) are identified. Consistent with previous factor analysis theory [23,36], when the number of factors is larger than the sample size the number and composition of the factors cannot be estimated.

**Figure 2:**
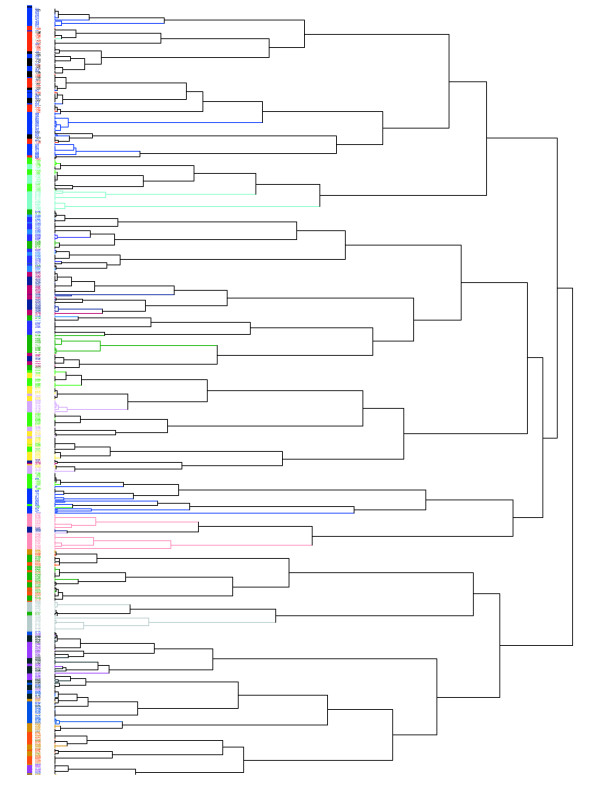
Hierarchical cluster plot of simulation with ten genotypes, 100 noise genes, and 20 factors. ρ = 0.4, effect size = 1, 2,...20. Blue, noise genes; other colors represent genes that should cluster together.

We then wanted to determine whether hierarchical cluster analysis would resolve this structure more clearly. We plotted the results in a dendrogram where each of the simulated factors are plotted with a separate color (Figure 2). No clear pattern of clustering was found. However, the clusters do form some 'kernels', so that biological knowledge of pathways could potentially be applied to interpret some of the groupings, as is common practice. However, the clear presence of noise genes throughout the cluster structure clearly makes interpretation difficult in cases where the true structure is unknown.

We then applied tight clustering to these data. When the correct number of clusters is specified, tight clustering does identify the structure of the 20 clusters. Each cluster consists of a subset of genes that belong to that cluster. Notably, it does not erroneously place noise genes into clusters. It performs less well in identifying the correct number of correlated genes failing to place 50% into clusters, instead classifying these correlated genes as noise. When a larger number of clusters (25) is specified, then there are some 'extra' clusters of noise genes and the clusters of genes are themselves not as distinct, that is 40 genes of the 600 are incorrectly grouped and 221 or 37% of the genes which should be in a cluster are designated as noise.

Overall, the simulation results indicate that focusing on a manageable number of possible factors with a measurable amount of difference in transcript abundance among lines can result in successful identification of factor structure, even when the number of genes examined is large relative to the sample size. We also find that the factor loadings can distinguish noise genes even in complex cases, although the factor structure can not be resolved clearly in those cases.

### Data analysis

#### Loci showing evidence for transcript variation

The GeneChip Drosophila Genome Array was used for this study and of the approximately 13,500 genes on the array, 7,886 showed expression on at least one array, and of these, 4,667 showed evidence for variation among genotypes. As we are studying covariation, we restricted our examination to this list of 4,667 loci (see Additional data file 1 [Supplementary Table 1]).

#### The immune pathway

**Figure 3:**
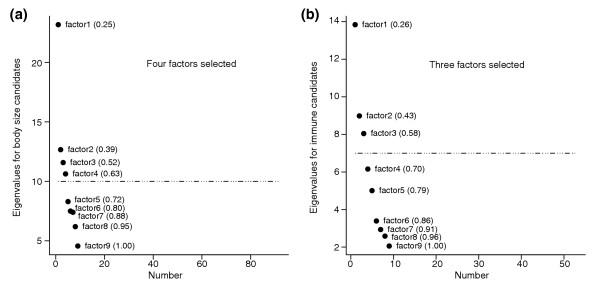
SCREE plots. The *x*-axis is the ordinal number of the eigenvalue and the *y*-axis is the magnitude of the eigenvalue. The number to the right of the plotted point indicates the cumulative variance explained as each factor is added. The dotted line indicates the cutoff point in the SCREE plot where there is a sharp drop off in the magnitude of the eigenvalues. The number of factors above the dotted line are the number retained for the factor analysis. **(a) **Body size, 92 genes; four factors are selected. **(b) **Immune, 53 genes; three factors are selected [36].

To provide an assessment of the performance of factor analysis on a set of well characterized genes, FlyBase [37] was queried for candidate genes involved in the immune pathway [38]. We compared the candidate genes to the list of 4,667 transcripts, and 54 genes were identified. Factor analysis on these transcript levels resulted in the identification of three factors (see Figure 3). Notably, the first factor contained all the lysogen genes present in the study, and the second factor contained all cecropins (Table 2). Factor-analysis groups co-regulated genes in a manner consistent with our understanding of the immune pathway. Hierarchical cluster analysis was also performed on this set of genes. As with the factor analysis, hierarchical cluster analysis found that the lysogen genes clustered together, as did the cecropins. However, determining the appropriate number of clusters was problematic and did not lead to any clear interpretation of the appropriate number of clusters. The final factor model for the immune genes included three factors, therefore we examined a k-means clustering analysis with three clusters for comparison. We found that 17/24 (71%) of the genes that loaded high on factor 1 were on the same cluster. This cluster also includes a few genes that loaded on factors 2 and 3. Genes that loaded high on the second and third factor were distributed among the remaining two clusters. The genes in these groups that did not load significantly on any factor were distributed among the three clusters.

#### Candidate loci for body size

We again queried FlyBase for a list of genes involved in body size determination and found 92 body size candidates in our list of 4,667 transcripts. Four factors were identified (Figure 3). The identification of covarying genes in the same factor was intriguing. Of particular note is the presence of loci that are contained within quantitative trait loci (QTL) for body size on factor 1: *Cdk4*, *trx*, *akt1*, *fru*, *Dr*, *mask*, *khc*, and *InR *[33].

In our analysis, we regressed transcript level for individual candidate genes on the body size phenotype. We found 2,892 genes significant at a nominal level of 0.05 (false discovery rate (FDR), 16%, see Table 3). At a more stringent nominal threshold of 0.01, there were 14 candidate genes for body size which showed significant association between transcript abundance and phenotypic variation for male body size (FDR of 7%). Of the QTL candidates, only *InR *showed significant association with the phenotype.

**Figure 4:**
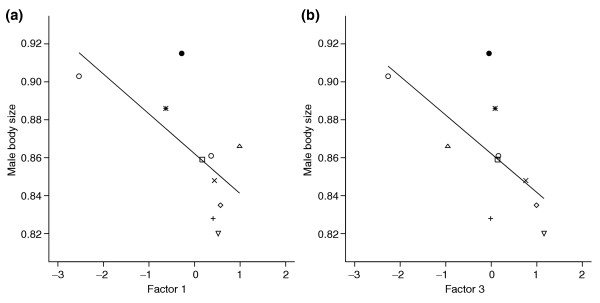
Regression plots. **(a) **A plot of factor 1 from candidate genes for body size on the *x*-axis and measured male body size on the *y*-axis. The solid line is the regression of factor 1 on measured male body size with an estimated slope of -0.021*y *with a standard deviation of 0.008 and is significantly different from zero (*P *= 0.04). Line crosses: open square 1136; open circle (top left), 611; open triangle 3743; plus sign, 4361; multiplication sign, 6177; open diamond, g785; inverted open triangle 8599; star 99105; solid circle, 1056; open circle (middle), 3637. **(b) **A plot of factor 3 from candidate genes for miRNA on the *x*-axis and measured male body size on the *y*-axis. The solid line is the regression of factor 3 on measured male body size with an estimated slope of -0.020*y *with a standard deviation of 0.008 and is significantly different from zero (*P *= 0.04). Symbols as in (a).

We then tested the hypothesis that the estimated factors were directly related to phenotypic variation in body size. In our analysis, we regressed the estimated factor (latent variable) on the phenotype body size for each genotype. The regression of factor 1 on body size showed evidence of an association between the factor and the phenotype of body size (*P *= 0.04, Figure 4a).

#### Targets of miRNAs

Of 535 putative miRNA targets [39], 203 were contained in our set of 4,667 gene transcripts. Factor analysis resulted in the identification of four gene factors (Table 4). The second factor contained four of the same genes as factor 1 for body size (*puc*, *Eh*, *mys*, *bon*) with 76 additional genes contained in this factor (loaded at 0.40 or greater). However, this factor was not associated with body size (*P *= 0.55). While some of the QTL candidates are also putative targets of miRNA regulation, (*Cdk4*, *trx*, *Dr*) these genes did not participate in this factor but were common to the factor identified by the third factor (see Table 4), for which 69 additional genes loaded. This third factor was negatively correlated with body size (*P *= 0.04, see Figure 4b). (*Cdk4*, *trx*, *Dr*) were not associated with body size in regressions between these individual genes and body size.

## Discussion

We applied factor analysis to high-dimensional microarray data. Using simulated data to estimate factors, we found that when correlation among genes is strong, the number of factors and their structure can be estimated, even in the case where genes unrelated to the factor structure (noise genes) are included. We also found that when noise genes were included hierarchical cluster analysis was unable to separate the noise genes from the signal, or to correctly identify the number of clusters. When the number of genes is large relative to the sample size, as is common in array studies, the number of factors and the genes belonging to each factor can still be identified, as long as the number of factors is less than the sample size. In contrast, hierarchical cluster analysis did not identify the number of clusters even when the number of clusters was smaller than the sample size.

We found that if the number of factors is larger than the number of genotypes, the majority (58%) of noise genes still do not load on any factor, while all but 26 of the 600 correlated genes do load on at least one factor. However, the correct association between individual genes and factors is lost. We conclude that while factor analysis is effective at separating the signal from the noise, the structure of the signal is not estimable. This is consistent with reports in the literature [23,36]. Using hierarchical clustering, the number and the structure of clusters is not recovered and noise genes are scattered throughout the cluster structure. Kernels of tightly correlated genes were visible, however, indicating that kernel identification is possible in cases where biological knowledge is present. Any separation of signal from noise is purely serendipitous.

These results are not unexpected as the mathematical properties of gene-expression data or the high dimensionality of the data lead to problems for any analysis. When the number of columns (in this case observations) is less than the number of rows (or variables), the matrix is considered ill conditioned [40]. Ill-conditioned matrices can cause problems for many types of statistical analysis and can lead to overfitting of predictive models, among other problems [41,42]. Some of the effects of the ill-conditioned matrices can be mitigated; however, the problem of an overdetermined system will always exist. An example is in multiple regression, where models with more variables than we have data can not be fit [43].

Tight clustering [27] represents a significant advance over hierarchical clustering in the estimation of cluster structure for microarray data. It provides a reasonable way of identifying most noise genes. In simple cases, however, the algorithm needs to have some flexibility when specifying starting values; that is, more rather than fewer clusters improve the chances of correctly identifying true clusters. The algorithm requires that the number of clusters be specified *a priori*. In contrast, in many simple cases the number and structure of factors can be recovered precisely using a factor analysis. In the most complex case examined (20 factors, 600 genes and 100 noise genes), when the number of clusters is correctly specified, tight clustering identifies 100% of the noise genes and the 20 clusters with correct genes within each cluster. However, it also incorrectly identifies 50% of genes with signal as noise genes. If too many clusters are specified (25), then the number of genes identified in clusters increases to 63%, although the correct structure is no longer maintained and 20% of the noise genes are incorrectly clustered. In contrast, factor analysis separates the signal from the noise, correctly identifying 96% of the noise genes as noise and 58% of the genes as having signal. In this complex case it is difficult to say whether factor analysis or tight clustering is 'better'. Both of these approaches clearly outperform hierarchical clustering, and they are complementary in their approach. If the primary goal is to separate the correlated genes from the uncorrelated genes then factor analysis performs better than tight clustering. If determining the number and structure of factors (coexpressed genes) is the goal, using both approaches and comparing findings will be reasonable.

Clear network structures can be identified with factor analysis and can then be followed up experimentally. Furthermore, unlike cluster analysis (hierarchical or tight), which provides no summary of the clusters into a single variable, the factor loading values are directly interpretable as the degree of participation of that locus in a factor. In contrast to cluster analysis, the factor loadings for genes give us information on the relative strength of a gene on a factor. We can use factor loadings to identify the most 'significant' genes on a factor and we can use the factor loadings to remove genes that are not contributing to any factor. As such, genes that all load highly on the same factor can be said to be coordinately expressed and putatively co-regulated, and noise genes can be identified as they will not load high on any factor. Unlike clustering, factor analysis allows genes to participate in several factors, thus reflecting biological reality more accurately.

Extensions of factor analysis have been developed and include allowing for nonlinearity [44,45] and the application of Bayesian approaches [46]. Factor analysis is itself a subclass of modeling techniques known as structural equation models [47] and the ongoing theoretical interest in these approaches allows for expansion of consideration to more than the present circumstance.

We focus our *Drosophila *analyses on a set of genotypes that are mated according to a round-robin design where the parental lines are natural variants. Analyses of such lines allows inferences about the underlying genetic contribution to variation and inferences about variation in pathways that occurs as a result of such natural genetic variation. By using candidate loci from reverse genetic and mutational projects, the importance of these loci in a broad context can be assessed. The factor analysis for the list of genes annotated as body size candidates resulted in the estimation of four factors. Factor 1 is of great interest as several of the genes that load on this factor - *Cdk4*, *trx*, *akt1*, *fru*, *Dr*, *mask*, *woc*, *Khc*, and *InR *- are contained in QTL for body size [33]. This overlap is exciting as the data from this QTL analyses are independent of our factor analysis. The identification of the same set of loci lends weight to the evidence that these loci are involved with the formation of body size in a natural population. Of these loci, only *InR *is directly correlated with body size. Given our limited knowledge of pathways for body size, it was exciting to note that two of the genes in this factor - *trx *and *M(2)21AB - *have been shown to interact [48]. In addition, the early sex-determination cascade genes *Sxl*, *tra*, and *tra2 *are all associated with a different factor (factor 2).

If the expression of multiple genes is regulated by a common underlying factor such as a transcription factor or a miRNA, and this regulation exhibits genetic variation, then we expect that the gene expression among these genes in a set of genotypes from a mating design will also be correlated. Factor analysis on putative targets of miRNA control revealed that putative targets of the same miRNA often occurred on the same factor. The number of targets for each miRNA was small, and so we cannot conclude that the coexpression is greater than we would expect by chance. Nevertheless, this is the first opportunity to examine the correlation structure of these putative targets of miRNAs. We found that of the 203 genes identified as miRNA targets, there was evidence for 188 participating in at least one of the four factors. While four genes on factor 1 for body size (*puc*, *Eh*, *mys*, and *bon*), and nine additional candidate body size genes (*Sh*, *Abd-B*, *trx*, *fng*, *qm*, *woc*, *Dr*, and *Cdk4*) are putative targets of miRNAs [39], the resulting miRNA factors are uncorrelated with the factors for body size. One of the miRNA factors is associated with the body size phenotype.

In summary, factor analysis, a technique developed to discover and model underlying mechanisms in complex social and psychiatric situations, seems to offer a reasonable middle ground for gaining understanding of coordinated gene expression. Our simulations show that when the number of underlying factors is larger than the sample size, it is not straightforward to recover the structure of the simulated data, although signal can be separated from noise. In contrast, target groups, even when the number of genes is large, can be used to identify several underlying regulatory mechanisms. In the case of body size for *Drosophila*, factor analysis offers an exciting opportunity to estimate gene networks, as relatively little is known about how genes involved in body size work together.

## Materials and methods

### Simulations

In trying to estimate the structure of gene networks, one of the first questions to be addressed is whether the number of genes contributing to each network affects the ability of the analysis to determine the structure of the network. Accordingly, we varied the number of genes examined in our simulations to explore this process (see Table 1). Gene networks were simulated as a set of correlated expression values. These networks represent genes affected by some common underlying biological process and the correlation structure that results from this biological connectedness is the concrete evidence of the network. In our simulations, for each network, a mean from a gamma distribution (γ) is drawn. A gamma distribution was chosen for the mean values across genes as this distribution has been shown to fit the distribution of transcript levels seen from genes on an array [49]. Genes in the network were simulated according to a multivariate normal distribution with correlation among genes indicated by the parameter (ρ). We looked at two levels of correlation ρ = 0.40 (weak) and ρ = 0.80 (strong). Genetic variation for individual genes within a network was simulated as a linear combination of the multivariate normal mean, a fixed genotypic effect and random noise. Genotypic effects for different lines differed in magnitude. The standard deviation was set to 1, and the standard normal was used, so that differences between lines can be directly interpreted as the size of the effect, that is the effect sizes are absolute and not relative to the magnitude of the gene expression (or amount of variation) for a particular gene. When the maximum difference among genotypes is less than 0.2 the effects are small, when differences are between 0.2 and 0.6 the effects are medium and when effects are larger than 0.8 they are relatively large [35]. Between networks the maximum difference of transcript abundance (effect size) among lines was allowed to vary.

Genes not participating in any network, were also simulated. These genes, uncorrelated to each other, are hereafter referred to as noise genes. For noise genes, the mean value for gene expression was drawn from a gamma distribution and variation from that mean was random and without regard to the genotype.

Concurrently, factor analysis on the data was performed and oblique rotations were used for estimating factor loadings [36]. We used the eigenvalues to determine the number of factors according to standard factor analytic approaches [36]. The plot of the eigenvalues, against the ordinal number for the eigenvalue (SCREE plot) is examined for the last substantial drop in the magnitude of the eigenvalues and a model with the same number of factors as the number of eigenvalues before the last substantial drop are retained [23,36]. Hierarchical cluster analysis was performed using a Ward distance. Tight clustering, a resampling-based approach to cluster analysis, was performed using software provided by [27].

### *Drosophila *lines

Isogenic lines of *Drosophila simulans *were made from flies caught in Wolfskill orchard [50] and crossed in a round-robin mating design as follows: the ten parental stocks (randomly sampled and independently derived from a large natural reference population) were crossed together in ten combinations to create heterozygous lines such that each parent was present twice, once as a dam and once as a sire. Crosses were (dam × sire): SIM6 × SIM11, SIM11 × SIM36, SIM36 × SIM 37, SIM37 × SIM43, SIM43 × SIM61, SIM61 × SIM77, SIM77 × SIM85, SIM85 × SIM99, SIM99 × SIM105, and SIM105 × SIM6. The resulting heterozygous genotypes were used for the experiment as follows: RNA from males was extracted and labeled 4-7 days post-eclosion [6,50]. Transcript level was estimated the average difference using MAS 5 [51]. Genes without positive signal on at least one array were removed from further consideration. The remaining genes were normalized to the chip median and log transformed. Genes lacking variation among genotypes cannot be meaningfully assessed for covariation. Accordingly, any gene that showed evidence (*P *≤ 0.2) for variation in transcript level across lines [50] or evidence for additive genetic effect (*P *≤ 0.05) [6] was considered in further analyses. Adult male flies were measured for body size [52].

### *Drosophila *data analysis

Candidate gene lists were developed using FlyBase queries. The miRNA targets were taken from Enright and colleagues [39]. The resulting lists were matched against the set of loci for which we had evidence of genetic variation in transcript abundance among lines.

In the first step of the data analysis, regression of transcript abundance for individual genes that were candidates for body size, or putative miRNA targets, was conducted to test the hypothesis that individual genes transcript levels were associated with body size. The average transcript abundance for each gene *i *within genotype *j *(*g*_*ij*_) was regressed on the average male body size (*Y*_*j*_) for each genotype *j *as follows:

*Y*_*j *_= μ + *g*_*ij *_+ ε_*ij*_.     (1)

The mean of all genotypes is μ and the random error is ε. Cluster analysis was performed on the set of immune genes. This was done using a hierarchical cluster analysis on the standardized values of gene expression. The results were plotted in a dendrogram.

Factor analysis on standardized values of gene expression, for each of the three lists (genes in the immune pathway, candidates for body size and putative miRNA targets) was conducted separately and factor loadings were estimated using an oblique rotation. The resulting set of eigenvalues was plotted in a SCREE plot, and the number of factors chosen such that the drop between eigenvalues was apparent, and a reasonable proportion of the variation was explained [36]. Each factor identified represents a gene network. Once the number of factors was identified, factor analysis was repeated for that fixed number of factors and loading values (the correlation between individual genes and the estimated factor structure) were estimated. All factor analyses were conducted in SAS (PROC FACTOR) using standard options, no special coding was required.

The effect of the factor upon the genotype is also estimable in the form of a factor value. Factor values were computed as a linear combination of the factor loading times the standardized value for the gene expression [18,23]. The factor value for genotype *j *(*GN*_*j*_) is an estimate of the impact of the network upon the genotype and is estimated as



where *g*_*ij *_is the expression value for gene *i *within genotype *j *and *l*_*i *_is the loading value estimated from the factor analysis for gene *i *across all genotypes. Differences in factor values represent differences between genotypes for the factor.

As all the genotypes are related in a mating design, we expect that differences in networks due to genetic variation which result in phenotypic variation should be detectable. Accordingly, the factor values were then regressed on the phenotype where

*Y*_*j *_= μ + *GN*_*j *_+ ε_*j *_    (2)

where *GN*_*j *_is the factor value for genotype *j*.

## Additional data files

The following additional data is available with the online version of this paper. Additional data file 1 lists the median values of gene expression for each of the ten lines (after normalization) for the 4,667 genes found to have some evidence of a line effect. Some basic annotation information from Affymetrix is also provided along with the feature number.

## Supplementary Material

Additional File 1 Line 1 through Line 10 medians for the 4667 genes considered in this analysis, after normalization (*line*1_*median*_) and standardization (*line*1_*median_st*_). Some basic annotation is also included as well as the Affymetrix feature number , Fall 2004). The median values of gene expression for each of the ten lines (after normalization) are provided for the 4,667 genes found to have some evidence of a line effect. These are labeled as line1_median-line10_median. Standardized values are also provided and labeled as line1_median _st-line1-_median_st. Some basic annotation information from Affymetrix is also provided along with the feature number. The chromosome number was inferred based upon the cytogenetic map position. Click here for file

## Figures and Tables

**Table 1:** Gene expression simulations

Number of genotypes	Number of factors	Number of genes		Correlation (ρ)	Effect size	Factors clearly identified	Proportion correct
						
		Noise	Each factor				
2	3	100	30	0.8	0.2,0.4,0.6	Y	1.00
							
					0.02,0.04,0.06	Y	1.00
				0.4	0.2,0.4,0.6	Y	0.84
					0.02,0.04,0.06	Y	0.66
							
2	3	1000	300	0.8	0.2,0.4,0.6	Y	1.00
				0.4	0.2,0.4,0.6	Y	0.66
							
10	3	100	30	0.8	1,2,3	N	0.81
				0.4	1,2,3	N	0.64
		0	30	0.8	1,2,3	Y	1.00
				0.4	1,2,3	N	0.63
							
10	20	100	30	0.4	1,2,...,20	N	-
					0.1,0.2,...,2	N	-

The number of genotypes simulated is given in the first column. The number of underlying latent factors is given in the second column, followed by the number of genes simulated that are not a part of any underlying factor. The number of genes on each factor is given next, and are simulated as a multivariate normal with pairwise correlation among genes within the factor of ρ. The mean for the first genotype is drawn from a gamma distribution, and the subsequent means were drawn from a multivariate normal, with standard deviation of one such that the maximum difference between the means can be interpreted as the genotypic effect size. Thus, for each underlying factor the simulated genotypic effect is the maximum difference in transcript abundance among genotypes for the first, second, and third factor, respectively. Factors are considered to be clearly identified if there is a substantial drop in the eigenvalues of the correlation matrix, and a reasonable proportion of the total variation is explained. The proportion correct is the proportion of genes correctly identified when setting the number of factors in the factor analysis to be the simulated number of latent factors. For the simulation with 20 latent factors we cannot compute the proportion correctly identified, as there are more simulated factors than possible factors.

**Table 2:** Factor analysis for candidate genes for immune function

	Factor 1		Factor 2		Factor3	
			
	Name	Load	Name	Load	Name	Load
1	dl	0.93	scrib	0.95	AttB	0.86
2	cact	0.86	CecA1	0.88	GNBP2	0.86
3	LysC	0.86	CecA2	0.85	CG16756	0.85
4	LysD	0.84	IM2	-0.84	CG8193	0.83
5	LysB	0.84	PGRP-SA	-0.78	Bc	0.74
6	LysE	0.83	CG5140	-0.76	Eip93F	-0.73
7	GNBP3	-0.81	IM1	-0.69	ref(2)P	0.72
8	tub	0.77	IM4	-0.69	CG2736	0.69
9	Tl	0.74	CG6214	0.66	CG3829	0.65
10	CG12780	0.74	CecC	0.63	PGRP-SC2	0.60
11	Mpk2	0.73	PGRP-SC1b	-0.59	IM1	-0.59
12	PGRP-LE	0.72	CG1643	-0.53	TepIV	0.53
13	Lectin-galC1	-0.72	PGRP-SD	0.52	CG6214	-0.46
14	LysS	0.70	TepIV	-0.51	Drs	-0.44
15	CG17338	0.67	cact	0.45	GNBP2	0.43
16	PGRP-SC1a	0.59	Anp	0.44	tub	0.43
17	IM4	-0.56	Lectin-galC1	0.43	Nos	0.42
18	Bc	-0.53	Tl	0.41	ref(2)P	0.50
19	ref(2)P	0.50				
20	PGRP-SC2	0.49				
21	ik2	-0.48				
22	BEST:GH02921	-0.48				
23	CG3066	0.46				
24	CG8193	0.45				

Factor analysis for candidate genes for immune function. There were 53 candidate genes and a three-factor model was fitted. The genes that loaded with a value greater than 0.40 are listed here. For each factor, the first column is the gene symbol name from [37] and the second column is the loading value for that gene. Genes are considered as loading 'significantly' if the absolute value of the loading value is ≥ 0.40. Genes are considered as loading 'high' if the absolute value of loading value is ≥ 0.70.

**Table 3:** Factor analysis for candidate genes for body size

	Factor 1			Factor 2			Factor 3			Factor 4		
				
	Name	Load	*p*_1_	Name	Load	*p*_1_	Name	Load	*p*_1_	Name	Load	*p*_1_
1	Cdk4	0.98	0.21	l(2)gl	-0.90	0.29	Jheh3	-0.97	0.02	dpp	0.79	0.25
2	Kr-h1	0.95	0.01	CkIIbeta	0.86	0.67	cdc2c	0.88	0.21	per	0.72	0.03
3	sqh	0.93	0.02	betaTub85D	-0.84	0.00	tgo	0.84	0.16	Top1	0.71	0.41
4	trx	0.93	0.26	lilli	0.80	0.09	jar	0.75	0.07	Jheh1	-0.69	0.00
5	babo	0.90	0.00	RpS3	0.78	0.10	Fs(2)Ket	0.75	0.02	wupA	0.65	0.11
6	Akt1	0.88	0.15	Cg25C	0.76	0.13	Sh	0.73	0.11	Fas2	-0.64	0.50
7	fru	0.85	0.13	tra	-0.75	0.20	corto	0.72	0.03	Eh	-0.62	0.12
8	vg	-0.83	0.00	CG17309	0.73	0.00	tok	-0.67	0.17	tkv	0.62	0.10
9	fng	0.78	0.56	dnc	-0.70	0.04	Jheh2	-0.65	0.00	tra	0.62	0.20
10	RpS13	0.72	0.05	mbt	0.69	0.18	woc	0.63	0.32	sbr	0.61	0.15
11	Dp	0.69	0.36	debcl	-0.69	0.08	Pi3K92E	0.57	0.12	Jheh2	-0.60	0.00
12	Mef2	0.68	0.06	RpS6	0.66	0.07	qm	-0.56	0.26	puc	0.59	0.01
13	Rac2	0.68	0.00	rut	0.64	0.07	aur	0.55	0.02	Nos	0.56	0.02
14	shot	0.65	0.00	ben	0.62	0.24	dare	-0.55	0.18	qm	0.56	0.26
15	puc	0.62	0.01	M(2)21AB	-0.59	0.17	Jheh1	-0.55	0.00	Dr	-0.56	0.19
16	M(2)21AB	0.61	0.17	bon	0.59	0.11	Nos	-0.52	0.02	Eip75B	0.53	0.00
17	Dr	0.60	0.19	l(3)mbt	0.58	0.01	hh	0.51	0.04	Pk61C	0.52	0.23
18	trk	-0.58	0.27	Pka-C1	0.57	0.55	Pk61C	0.50	0.65	ftz-f1	0.51	0.08
19	Eip75B	0.58	0.00	Eip63E	-0.57	0.15	fru	0.50	0.11	CG11910	-0.49	0.02
20	fru	0.56	0.11	tkv	0.54	0.10	M(2)21AB	0.49	0.17	prod	-0.47	0.39
21	mask	0.56	0.07	rok	-0.54	0.16	mask	0.49	0.07	ninaE	-0.47	0.19
22	woc	-0.56	0.32	per	0.52	0.03	how	0.49	0.41	dnc	0.45	0.04
23	Dot	-0.53	0.11	Sxl	0.50	0.15	neb	0.48	0.74	robl	-0.45	0.38
24	Khc	0.53	0.14	neb	0.48	0.74	Egfr	-0.47	0.24	InR	0.44	0.01
25	ade2	0.52	0.01	Eh	0.48	0.12	RpS3	-0.47	0.10	vg	0.42	0.00
26	Tsc1	-0.52	0.04	prod	-0.47	0.39	mys	-0.46	0.11	Pka-C1	-0.41	0.55
27	Fas2	0.51	0.50	wupA	0.47	0.11	robl	-0.44	0.38	Khc	0.41	0.14
28	l(3)mbt	-0.50	0.01	shot	0.46	0.00	Kr-h1	-0.42	0.01	corto	0.40	0.03
29	Dfd	-0.49	0.19	Pk61C	-0.44	0.23	Tor	0.41	0.20			
30	mys	-0.49	0.11	Ddc	-0.43	0.19						
31	Sh	-0.47	0.11	tra2	-0.42	0.24						
32	how	0.47	0.41	InR	0.42	0.01						
33	Iswi	-0.46	0.13	Pi3K92E	0.42	0.12						
34	InR	0.45	0.01	hh	0.40	0.04						
35	ben	0.44	0.24	Tor	0.40	0.20						
36	neb	0.43	0.74									
37	Top1	-0.41	0.41									
38	tgo	-0.40	0.16									

Factor analysis for candidate genes for body size. There were 92 candidate genes and a four factor model was fit. The genes that loaded with a value greater than 0.40 are listed here. For each factor, the first column is the gene symbol name from [37] and the second column is the loading value for that gene. Genes are considered as loading 'significantly' if the absolute value of the loading value is greater than or equal to 0.40. Genes are considered as loading 'high' if the absolute value of the loading value is greater than or equal to 0.70. The third column for each factor is the *p*-value for the individual gene expression value regression on male body size (*p*_1_).

**Table 4:** Factor analysis for putative targets of miRNAs

	Factor 1			Factor 2			Factor 3			Factor 4		
				
	Name	Load	*p*_1_	Name	Load	*p*_1_	Name	Load	*p*_1_	Name	Load	*p*_1_
1	kel	-0.90	0.05	CG5805	-0.92	0.04	CG7995	0.89	0.01	tws	0.80	0.06
2	CG6327	0.89	0.18	Cirl	0.89	0.23	CG4710	0.89	0.07	CG3689	0.78	0.14
3	CG18812	0.88	0.00	CG9245	0.82	0.05	CG4851	0.87	0.19	CG3811	0.77	0.11
4	Cyp314a1	-0.88	0.06	l(2)03709	0.82	0.13	Rpn6	0.87	0.12	CG11883	0.77	0.03
5	Gclc	0.87	0.01	Eh	0.82	0.12	up	-0.84	0.24	CG10809	0.74	0.34
6	sima	0.86	0.11	fz	0.81	0.13	Pkc98E	0.82	0.42	CG11128	0.74	0.17
7	CG9924	0.85	0.17	CG6330	0.80	0.11	CSN4	0.80	0.01	CG3961	0.72	0.15
8	CrebA	0.84	0.00	Atpalpha	0.79	0.38	cpo	0.72	0.41	CG5087	0.72	0.06
9	Mbs	-0.84	0.04	Surf4	0.79	0.29	drongo	0.72	0.12	unc-13	-0.70	0.07
10	fax	0.79	0.03	sano	0.79	0.02	fng	0.71	0.56	CG12424	0.70	0.16
11	RhoGEF2	0.78	0.11	CG12424	-0.78	0.40	G-oalpha47A	0.70	0.05	CG13344	0.70	0.03
12	CG9664	-0.78	0.34	CG4911	0.78	0.48	PFgn0025879	0.69	0.26	Sap47	0.69	0.05
13	CG2991	0.77	0.12	JhI-21	-0.77	0.34	Cka	-0.69	0.12	CG6325	0.69	0.29
14	CG8602	-0.76	0.04	CG9339	0.77	0.09	Ptp99A	0.68	0.07	CG8475	0.68	0.09
15	BicD	0.76	0.07	CG18604	0.76	0.02	cenG1A	0.67	0.22	CG5625	0.67	0.04
16	CG8954	0.76	0.29	G-oalpha47A	-0.76	0.02	Ef1gamma	0.67	0.18	PFgn0025879	0.67	0.26
17	dock	0.75	0.08	CG11266	0.76	0.06	CG4452	0.67	0.03	CG5039	0.67	0.01
18	CG10077	0.75	0.03	CG15628	0.75	0.11	CG3764	0.66	0.05	CG3534	-0.67	0.14
19	CG9381	-0.73	0.33	AP-47	0.73	0.15	CG5853	0.66	0.00	Pkc53E	0.65	0.15
20	Mbs	0.72	0.11	CG14762	0.73	0.05	ed	0.65	0.07	CG17646	0.65	0.13
21	BG:DS04929.1	0.71	0.03	CG4963	0.73	0.11	trx	0.65	0.26	CG11178	0.64	0.07
22	bon	0.70	0.11	Eip71CD	0.68	0.09	Cyp18a1	0.64	0.17	CG1814	-0.63	0.00
23	CG9413	0.70	0.60	CG7956	0.67	0.11	SoxN	0.63	0.03	CG14989	0.62	0.07
24	CG6282	-0.69	0.16	ple	0.66	0.17	eIF-5A	0.63	0.23	Sh	0.61	0.11
25	puc	0.69	0.01	CG9297	0.66	0.03	Atpalpha	0.63	0.02	CG9828	0.59	0.20
26	Mkp3	0.68	0.09	CG11198	0.66	0.01	Dr	0.62	0.19	pdm2	-0.58	0.20
27	CG4841	0.68	0.19	Cyp49a1	0.65	0.00	CG3961	0.62	0.15	CG6803	0.58	0.01
28	CG5886	0.67	0.05	gish	0.65	0.04	lack	0.61	0.08	Ptp99A	-0.56	0.07
29	CG9934	0.67	0.42	wdp	-0.65	0.01	G-oalpha47A	-0.61	0.02	Abd-B	0.55	0.06
30	Rab6	0.66	0.09	Pdi	0.65	0.14	CG16971	0.60	0.17	ana	0.55	0.03
31	CG7492	-0.65	0.29	sdk	0.63	0.34	Cdk4	0.60	0.21	CG6199	-0.54	0.26
32	CanA-14F	0.65	0.01	CG4484	-0.63	0.23	CG18854	0.60	0.06	Nmda1	0.54	0.34
33	CG10338	-0.65	0.03	nmdyn-D7	0.62	0.08	aop	0.60	0.01	CG9265	-0.53	0.13
34	CG8617	-0.65	0.22	scrt	-0.61	0.17	tws	0.59	0.00	CG10494	0.51	0.26
35	CG6064	0.64	0.01	mys	-0.61	0.11	wdp	0.58	0.01	CG9638	0.50	0.05
36	fkh	0.64	0.13	CG11537	0.61	0.33	CG1441	0.57	0.33	RhoGAPp190	0.50	0.00
37	Cka	0.64	0.12	CG8104	0.60	0.07	Cyp49a1	-0.57	0.18	CG4452	0.48	0.03
38	Mkp3	0.62	0.01	Mbs	0.60	0.11	CG17646	0.57	0.02	CG6554	-0.47	0.38
39	trx	0.62	0.26	CG5853	-0.60	0.00	CG4841	0.57	0.19	CG18375	-0.46	0.00
40	CG13586	0.62	0.25	tsl	0.59	0.00	CrebA	0.55	0.10	woc	0.45	0.32
41	amon	-0.61	0.21	UbcD2	0.58	0.21	Ef1alpha100E	0.54	0.08	CG8791	0.45	0.03
42	osp	0.60	0.01	ytr	0.58	0.02	Hr39	0.52	0.21	BicD	-0.45	0.07
43	Trn	0.60	0.02	G-oalpha47A	0.58	0.05	woc	-0.50	0.32	dco	0.44	0.02
44	CG7283	0.60	0.00	CG3800	0.56	0.52	vri	0.50	0.00	amon	0.44	0.21
45	Eip93F	0.59	0.06	Tsf2	0.56	0.21	CG16953	-0.50	0.10	CG1441	-0.44	0.33
46	CrebA	0.59	0.10	BcDNA:LD23587	0.56	0.20	dco	-0.49	0.02	CG9297	-0.43	0.03
47	Ptp4E	0.59	0.79	Nmda1	-0.54	0.34	puc	0.49	0.01	CG13586	-0.43	0.25
48	BcDNA:LD32788	0.58	0.13	Ubc-E2H	-0.53	0.00	CG8475	0.48	0.09	CG9664	-0.42	0.34
49	Hr39	0.56	0.21	Ac3	0.52	0.23	CG8602	0.48	0.04	scrt	0.41	0.17
50	ed	0.56	0.07	CG15658	-0.51	0.10	CG12424	-0.47	0.16	Cf2	0.41	0.47
51	CG11099	0.56	0.05	bon	0.50	0.11	Asph	0.47	0.02	insc	0.41	0.14
52	sdk	-0.56	0.34	sbb	0.49	0.10	BcDNA:LD32788	0.46	0.13	CG4484	0.40	0.23
53	cenG1A	0.55	0.22	SoxN	-0.49	0.03	Cf2	-0.46	0.47	Tsf2	0.40	0.21
54	Vha16	0.54	0.01	aop	-0.48	0.01	CG9638	0.46	0.05			
55	BcDNA:LD23587	0.54	0.20	CG3764	0.47	0.05	CG14762	-0.46	0.05			
56	CG18604	0.54	0.02	CanA-14F	0.47	0.01	CG11266	0.45	0.06			
57	Trn	0.52	0.07	CG16953	0.47	0.10	Eip93F	-0.44	0.06			
58	CG15236	-0.52	0.06	CG15236	-0.47	0.06	CG9413	0.44	0.60			
59	fng	0.52	0.56	Trn	0.45	0.02	Abd-B	0.44	0.06			
60	did	-0.51	0.18	Cka	-0.45	0.12	CG6707	0.43	0.75			
61	CG11537	0.51	0.33	Ptp4E	0.44	0.79	BcDNA:LD21720	-0.43	0.12			
62	CG10494	-0.51	0.26	BG:DS04929.1	0.44	0.03	Cyp49a1	-0.43	0.00			
63	CG3534	-0.50	0.14	CG18854	0.44	0.06	CG8791	-0.42	0.03			
64	pdm2	-0.50	0.20	vri	-0.44	0.00	Sh	-0.42	0.11			
65	CG8451	-0.50	0.06	CG9924	-0.43	0.17	CG15658	0.42	0.10			
66	Eip71CD	-0.49	0.09	dco	0.43	0.02	l(2)03709	0.41	0.13			
67	gish	0.49	0.04	lack	0.43	0.08	CG6803	0.41	0.01			
68	Aef1	-0.49	0.11	CG8791	-0.43	0.03	CG15628	0.41	0.11			
69	GLaz	0.48	0.11	CG9664	0.43	0.34	Cyp310a1	-0.41	0.00			
70	dco	0.47	0.02	CG5087	-0.43	0.06	BicD	-0.41	0.07			
71	sbb	0.45	0.10	CG14989	-0.42	0.07	tsl	-0.41	0.00			
72	BcDNA:LD21720	0.45	0.12	CG6064	0.42	0.01	CG7283	0.40	0.00			
73	CG1814	0.44	0.00	CG18375	0.42	0.00						
74	CG6199	0.43	0.26	ATPCL	0.41	0.00						
75	CG4851	-0.42	0.19	puc	-0.41	0.01						
76	CG16971	0.41	0.17	CG9638	-0.41	0.05						
77	Sap47	0.40	0.05	CG6707	0.41	0.75						
78	M(2)21AB	0.40	0.17	CG17646	-0.41	0.02						
79	CG4452	0.40	0.03	Atet	0.40	0.08						
80				Cf2	-0.40	0.47						

Factor analysis for putative targets of miRNAs. There were 203 candidate genes and a four-factor model was fit. The genes that loaded with a value greater than 0.40 are listed here. For each factor, the first column is the gene symbol name from [37] and the second column is the loading value for that gene. Genes are considered as loading 'significantly' if the absolute value of the loading value is ≥ 0.40. Genes are considered as loading 'high' if the absolute value of loading value is ≥ 0.70. The third column for each factor is the the *P*-value for the individual gene expression value regression on male body size (*p*_1_).
